# Identification and Characterization of Small Noncoding RNAs in Genome Sequences of the Edible Fungus *Pleurotus ostreatus*

**DOI:** 10.1155/2016/2503023

**Published:** 2016-09-15

**Authors:** Jibin Qu, Mengran Zhao, Tom Hsiang, Xiaoxing Feng, Jinxia Zhang, Chenyang Huang

**Affiliations:** ^1^Institute of Agricultural Resources and Regional Planning, Chinese Academy of Agricultural Sciences, Beijing, China; ^2^Key Laboratory of Microbial Resources, Ministry of Agriculture, Beijing, China; ^3^School of Environmental Sciences, University of Guelph, Guelph, ON, Canada; ^4^College of Computer Science and Software Engineering, Shenzhen University, Shenzhen, Guangdong, China; ^5^Shenzhen Micro & Nano Research Institute of IC and System Applications, Shenzhen, Guangdong, China

## Abstract

Noncoding RNAs (ncRNAs) have been identified in many fungi. However, no genome-scale identification of ncRNAs has been inventoried for basidiomycetes. In this research, we detected 254 small noncoding RNAs (sncRNAs) in a genome assembly of an isolate (CCEF00389) of* Pleurotus ostreatus*, which is a widely cultivated edible basidiomycetous fungus worldwide. The identified sncRNAs include snRNAs, snoRNAs, tRNAs, and miRNAs. SnRNA U1 was not found in CCEF00389 genome assembly and some other basidiomycetous genomes by BLASTn. This implies that if snRNA U1 of basidiomycetes exists, it has a sequence that varies significantly from other organisms. By analyzing the distribution of sncRNA loci, we found that snRNAs and most tRNAs (88.6%) were located in pseudo-UTR regions, while miRNAs are commonly found in introns. To analyze the evolutionary conservation of the sncRNAs in* P. ostreatus*, we aligned all 254 sncRNAs to the genome assemblies of some other Agaricomycotina fungi. The results suggest that most sncRNAs (77.56%) were highly conserved in* P. ostreatus*, and 20% were conserved in Agaricomycotina fungi. These findings indicate that most sncRNAs of* P. ostreatus *were not conserved across Agaricomycotina fungi.

## 1. Introduction

*Pleurotus ostreatus *(Jacq.: Fr.) Kumm. (Dikarya; Basidiomycota; Agaricomycotina; Agaricales) is an important commercially available edible fungus worldwide, and it is the most popular edible mushroom in Northern China. This fungus can grow easily on a variety of organic substrates, including agricultural wastes [1, 2]. In addition to its delicious taste and nutritional value [3], this mushroom also has health-promoting effects [4]. Furthermore, it is tolerant of a wide temperature range during the cultivation [5]. Because of its wide substrate utilization, it is a good model for the study of lignin biodegradation [6] and environmental adaptation.

Noncoding RNAs (ncRNAs) producing functional RNA products instead of proteins [7] are widely expressed in both prokaryotes and eukaryotes [8–10]. For example, around 98% of transcriptional output in human is ncRNA [11]. NcRNA families are grouped into structural ncRNA and regulatory ncRNA based on their structure and function [9]. The structural ncRNA includes transfer RNA (tRNA) and ribosomal RNA (rRNA), as well as other small but stable noncoding RNAs, such as small nuclear RNAs (snRNAs), small nucleolar RNAs (snoRNAs), Ribonuclease P (RNase P), mitochondrial RNA processing (MRP) RNA, signal recognition particle (SRP) RNA, and telomerase RNA. Regulatory ncRNAs include microRNAs (miRNAs) and long ncRNAs (lncRNAs) [12]. These ncRNAs play important roles in splicing [13], transcription [14], translation [15], and chromatin architecture [16], and many ncRNAs are associated with diseases [17–24].

Recently, ncRNAs have been identified by experimental and computational methods in several fungi [10, 25–28]. But so far, there have been few studies related to ncRNA in basidiomycetes and even fewer for edible mushroom. Apart from rRNAs and a few tRNAs, no other ncRNAs have been annotated and characterized in the* P. ostreatus* genome. In this research, we sequenced the genome of a strain of* P. ostreatus* and identified small ncRNAs (sncRNAs) in the genome assembly. Then the distribution of genomic loci of these sncRNAs was characterized to describe the preferential locations of different sncRNAs. Lastly, we analyzed the evolutionary conservation of these sncRNAs among basidiomycetous fungi.

## 2. Materials and Methods

### 2.1. Strains and Culture Conditions

The* Pleurotus ostreatus* dikaryotic strain, CCMSSC00389, is widely cultivated in China and is preserved in the China Center for Mushroom Spawn Standards and Control, Institute of Agricultural Resources and Regional Planning, Chinese Academy of Agricultural Sciences. From this strain, the two nuclear types were separated to constituent monokaryons by dedikaryotisation as follows: the dikaryon was grown in 10 cm diameter Petri dishes containing 25 mL of potato dextrose agar (PDA) at 25°C for 6-7 days. Mycelia (1 g) were collected from the growing margins of the plate and suspended in 2% lytic enzyme (Guangdong Institute of Microbiology, China) and 0.6 mol/L mannitol and incubated at 30°C for 4 h. The resulting protoplasts were washed twice with 0.6 mol/L mannitol and placed (dissolved = broken up) in mannitol solution. The protoplast suspension was spread onto malt-yeast-glucose (MYG) medium and incubated at 25°C for 4-5 days. Monokaryons were identified by microscopy among the regeneration clones by lack of clamp connections and further confirmed by mating to produce dikaryotic hyphae with clamps connections. A single monokaryon of each nuclear type was randomly selected and sequenced and named CCEF00389 and CCEF00389_9.

### 2.2. Isolation of Genomic DNA

Genomic DNAs of the two monokaryons were extracted using a DP305-Plant Genome Extraction Kit (Tianjin, China). The purity and quality of the genomic DNA were determined through spectrophotometry and electrophoresis on a 1.0% agarose gel and sequenced using the Illumina HiSeq 2500. The raw data were generated by paired-end and mate-pair sequencing with different insert sizes. Strain CCEF00389 used a whole genome* de novo* sequencing strategy with average coverage of over 300x. Three libraries were constructed for 100 bp paired-end (300 bp insert size) and mate-pair sequencing (3 kbp and 8 kbp insert length).

### 2.3. Transcriptomic Data

Mycelia of the same strain were inoculated on the DifcoTM Potato Dextrose Agar plates with cellophane at 25°C for four days and were subjected to heat stress at 37 centigrade for different time (0, 0.5, 1, and 1.5 h). The mycelia through different treatment were collected, respectively, for RNA extraction. The RNA samples were then sequenced with Illumina HiSeq 2500. One library for each time point was constructed for 100 bp paired-end (300 bp insert size) sequencing. The raw data were assembled to the transcriptome with* de novo* assembler TRINITY [29].

### 2.4. Genome Assembly and Annotation

Raw reads were first trimmed by stripping the adaptor sequences and ambiguous nucleotides using SeqPrep (https://github.com/jstjohn/SeqPrep) and Sickle (https://github.com/najoshi/sickle). Reads with quality scores less than 20 or “N” more than 10% or lengths below 25 bp were removed. The cleaned reads were assembled using the tools PLATANUS [30] and L_RNA_Scaffolder [31] with* de novo* assembly guided by the assembled transcriptome. Gene models in the genome assembly of* P. ostreatus* were predicted using BRAKER1 [32]. The protein-coding genes were then confirmed using BLAST+ (version 2.2.31) against public databases, including the NCBI nonredundant database (NR) database, the Refseq database of fungi, ESTs of* P. ostreatus* PC15 (http://genome.jgi.doe.gov/pages/dynamicOrganismDownload.jsf?organism=PleosPC15_2), the predicted protein models of 134 basidiomycetous species in JGI website (http://genome.jgi.doe.gov/basidiomycota/basidiomycota.info.html), and the transcriptome of CCEF00389. The predicted gene models were then classified according to Gene Ontology (GO) [33] with homologous sequences in the NR database and also annotated by their protein domains using InterProScan [34].

### 2.5. SncRNA Detection

Small ncRNAs were first identified by aligning Rfam sequences to our genome assembly using BLAST+ and Infernal (version 1.0.3). These sncRNAs included snRNAs and snoRNAs. tRNAs were predicted with tRNAscan-SE (version 1.3.1) [35]. miRNAs were detected by alignment of Rfam miRNA sequences (RF00003) to our genome assembly with BLASTn, with the *e*-value cutoff 1*e* − 3 and the word size 19.

### 2.6. Nucleotide Sequence Accession Number

This whole genome shortgun project has been deposited at DDBJ/EMBL/GenBank (http://www.ncbi.nlm.nih.gov/) under the accession number MAYC00000000 (the project accession number PRJNA327267).

## 3. Results and Discussion

### 3.1. Genome Information of CCEF00389

**Figure 1:**
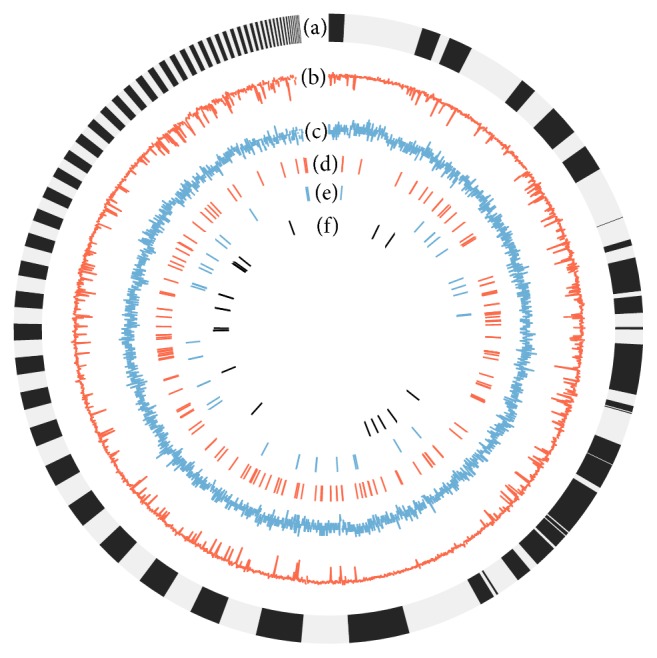
The ideogram showing the genomic features of* P. ostreatus*. (a) Scaffolds longer than 10 kbp. (b) GC content: the percentage of G+C in 10 kbp nonoverlapping windows. (c) Gene density: the number of genes in 10 kbp nonoverlapping windows. (d) Distribution of tRNAs. (e) Distribution of miRNAs. (f) Distribution of snRNAs, snoRNAs, and others.

A 34.9-Mb genome assembly was obtained by assembling approximately 81 million Illumina reads (~300x coverage) (Table 1 and Figure 1). Gene prediction from all scaffolds of the assembled genome and transcriptomic data generated 13,438 gene models. The genome size, number of predicted genes, and the basic information of predicted genes are very similar as those of related edible Agaricales, such as* Volvariella volvacea* [36],* Agaricus bisporus* [37], and* Flammulina velutipes* [38] (see Supplement Table 1 in Supplementary Material available online at http://dx.doi.org/10.1155/2016/2503023). Gene Ontology (GO) annotations were found for 6,566 proteins (48.9%) with homologous sequences in the NR database. In addition, 9,931 (73.9%) of all predicted genes can be annotated by their protein domains by InterProScan.

### 3.2. Identification of sncRNAs in* P. ostreatus*

#### 3.2.1. sncRNAs from Rfam 11: snRNAs, snoRNAs, and Other sncRNAs

The spliceosome contains five essential snRNAs: U1, U2, U4, U5, and U6 [39]. Four of them were identified in the CCEF00389 genome assembly: U4 and U5 exhibited a precise genomic location, while U2 and U6 had several candidate locations in the genome assembly (Table 2). To find the U1 genomic locus in the genome, we downloaded the U1 sequences of all species from Rfam and the U1 sequences of fungi from NCBI to be used as query sequences to search for homologues in the CCEF00389 genome using BLASTn. Interestingly, U1 was not found in this genome assembly, even after extensive searching with sequences from other fungi. Furthermore, U1 was not found in genome assemblies of other basidiomycetous fungi including* Agaricus bisporus *[37],* Coprinopsis cinerea *[40],* Flammulina velutipes *[38],* Schizophyllum commune *[41],* Pleurotus ostreatus PC15 *[42],* Volvariella volvacea *[36],* Laccaria bicolor *[43],* and Ustilago maydis *[44]. This implies that if snRNA U1 of basidiomycetes exists, it has a sequence that varies significantly from other organisms.

Small nucleolar RNAs (snoRNAs) guide chemical modifications of other cellular RNAs, including rRNAs, tRNAs, and snRNAs. There are two major classes of snoRNA in eukaryotic cells: the C/D box snoRNAs, which are associated with methylation, and the H/ACA box snoRNAs, which are associated with pseudouridylation [10, 45]. Seven snoRNAs were identified in the CCEF00389 genome assembly: three of them were of Rfam class snoZ13_snr52, and each of the others is belonging to Rfam class snosnR60_Z15, SNORD24, Afu_455, and SNORD46, respectively. There were also six other sncRNAs in the Rfam searching result: one RNase_MRP RNA and five Hammerhead ribozymes (type 3).

#### 3.2.2. tRNA

A transfer RNA (tRNA) is adaptor RNA molecule that serves as the physical link between the mRNA and protein [46], so it is a necessary component of translation and essential for life. However, the number of tRNAs in the genome assemblies of different organisms varies tremendously [47–49]. In the genome assembly of CCEF00389, we identified 185 tRNAs with length from 71 to 144 nt with their loci and anticodons shown in Supplement Table 2.

#### 3.2.3. miRNA

A micro-RNA (miRNA) is a small noncoding RNA molecule about 22 nucleotides in length, which functions in RNA silencing and posttranscriptional regulation [50]. The miRNAs have been identified in the genome assemblies of most eukaryotic organisms and are very abundant in many of them [51–54]. There were only 46 mature miRNAs identified in the CCEF00389 genome assembly by BLASTn, with lengths from 19 to 23. The most important factor in uncovering putative miRNAs was the parameter “word size” of BLASTn. If this parameter was set to 20, many fewer matches (only 10) were found. As we know, some miRNAs have a variation of 1-2 nt at the end (often 3′ end) [51]. And a probable reason of the lack of miRNAs in this genome assembly is that there is no currently available miRNA database for basidiomycetes. Compared with the known miRNAs, the sequences are not evolutionarily conserved.

### 3.3. Distribution of snRNAs in the CCEF00389 Genome Assembly

Most sncRNAs are located in noncoding regions of the genome, including introns, UTRs, and intergenic regions. The location of ncRNA might be associated with its function. For example, the ncRNAs in UTRs and intergenic regions may play cisregulatory roles regulating their adjacent genes, and/or transregulatory roles elsewhere in the genome [55]. And the ncRNAs in introns could regulate gene expression through transcriptional gene silencing (TGS) pathways [56, 57] and posttranscriptional gene silencing pathways [58–60].

We wanted to identify the sncRNAs to locus and characterize their distribution. The UTR regions of the CCEF00389 genome assembly were not identified, so the distribution of sncRNAs located outside the ORFs (from the start codon to the stop codon) was defined quantifiably as the distance to the nearer gene boundary (start/stop codon). The UTR regions usually lie within 2000 bp of gene boundary [61], and it can be assumed that the less the distance to gene boundary, the greater the possibility to be located in UTR [62].

**Figure 2:**
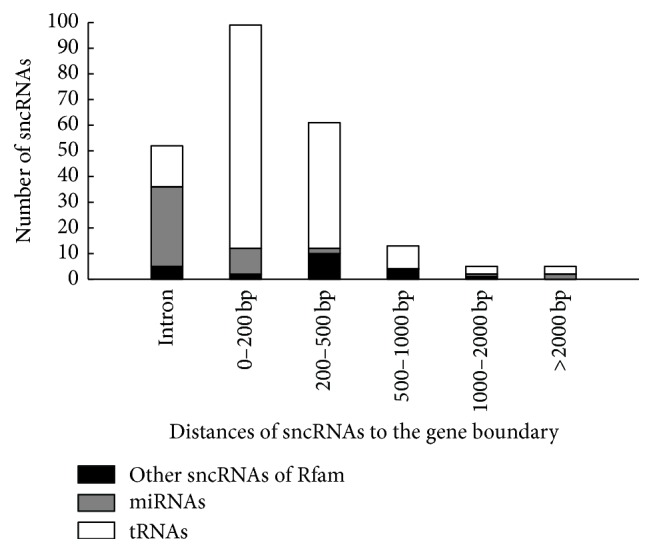
Distance of sncRNAs to the gene boundary (outside a start or stop codon).

For the three kinds of sncRNAs (tRNAs, miRNAs, and other sncRNAs from Rfam), the distribution is shown in Figure 2.

All detected snRNAs were located within 1,000 bp of a gene boundary. Among them, U5 was located 959 bp from a gene boundary, and the other snRNAs were located within 536 bp of gene boundaries. It is highly likely that all the snRNAs are located in pseudo-UTR regions of this genome. A similar distribution of snRNAs was found in the filamentous fungus* Trichophyton rubrum* [25]. For the snoRNAs, Hammerhead RNAs, and the RNase MRP, they were located diversely in the genome assembly: within 1031 bp of the gene boundary and in introns (5 out of 13) (see Table 2).

Most tRNAs (136 out of 167, 81.44%) located within 500 bp of gene boundary; this means that tRNAs distributed mainly in pseudo-UTR regions. There are also 16 tRNAs (9.58%) located in introns. For details, see Supplement Table 3.

As many as 67% (31 out of 46) of miRNAs in the CCEF00389 genome assembly located in introns, which are usually regulated together with their host genes [63, 64]. Two miRNAs, miR1171 and miR3948, located at a distance of more than 2000 bp away from an ORF, were intergenic (see Table 3).

### 3.4. Evolutionary Conservation of sncRNAs in* P. ostreatus*

In order to analyze the evolutionary conservation of sncRNAs in* P. ostreatus*, all identified sncRNAs were then aligned to the genomes of other fungi, including six* P. ostreatus*-related Agaricomycotina fungi:* Agaricus bisporus* [37],* Coprinopsis cinerea *[40],* Flammulina velutipes *[38],* Schizophyllum commune *[41],* Pleurotus ostreatus *PC15 [42],* Volvariella volvacea *[36], and finally* Ustilago maydis *[44] which is a basidiomycete, but basal to the Agaricomycotina.

Only 10 of these sncRNAs were also identified in all selected basidiomycetes, and all these conserved sncRNAs were miRNAs. This means that only a small part of miRNAs are conserved because of the low rate of evolution [65]. Only 5.9% (15 out of 254) of sncRNAs in the CCEF00389 genome assembly had homologues in* Ustilago maydis*. Most sncRNAs identified in Agaricomycotina fungi do not have homologues in other groups of fungi. There were 51 of these sncRNAs also identified in all six genomes of Agaricomycotina fungi, and 74 of these sncRNAs were also identified in at least five genomes of Agaricomycotina species. Moreover, the sequence identities of the matches were above 80%. This suggests that many sncRNAs are highly conserved among Agaricomycotina fungi. These conserved sncRNAs included the snRNA U2 (4 candidates) and U6 (3 candidates), 10 miRNAs (miR2673a, miR2673b, and miR-4968-3p), and 57 tRNAs (see Supplement Table 4). In some previous researches, the microRNAs miR2673 and miR-4968-3p were found to have many target genes in many species [66, 67] and may regulate some targets [68, 69]. MicroRNA miR2673 was also be found to have stable structure and be conserved across plant species [70].

**Figure 3:**
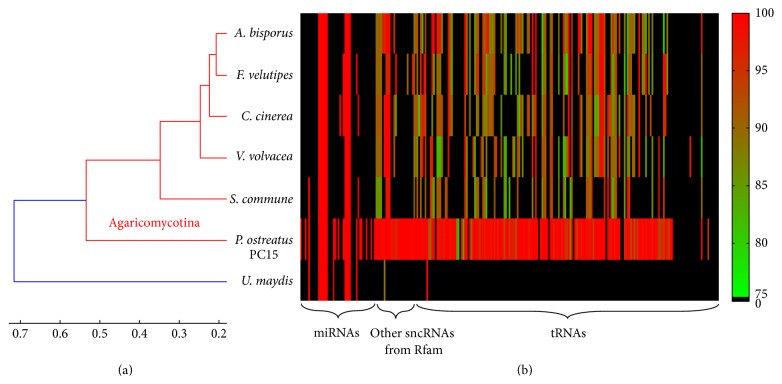
Similarity clustering based on sequence identities of sncRNAs between CCEF00389 and seven other basidiomycetous fungi: (a) the hierarchical clustering tree; (b) the heat map of identities; the black color means no matches at the cutoff 1*e* − 3 (1*e* − 1 for miRNAs, because the length of their sequences is short).

To compare the sequence similarity between sncRNAs of CCEF00389 and their homologues in selected fungi, a hierarchical clustering was performed to partition the different fungi based on the sequence identities. In the hierarchical clustering method, the Spearman correlation coefficient of sequence identities of all sncRNAs (if no matches were found, the identity was set to zero) was selected to define the dissimilarity between organisms.  Figure 3 shows the result of clustering. It is clear that the homologues of sncRNAs of* Pleurotus ostreatus *PC15 were most similar to sncRNAs of CCEF00389, because they belong to the same species. There were 77.56% (197 out of 254) of matched sncRNAs with sequence identities above 81.65%. For the other five organisms, the clustering results basically reflected the currently accepted phylogenetic placement of these species [71].

## 4. Conclusions

The CCEF00389 genome assembly is the first released draft genome of a strain of* P. ostreatus *in China. The genome size, number of genes, and some protein families were in accordance with the released genome of PC15, which is a North American strain of* P. ostreatus*. In the CCEF00389 genome assembly, we detected 254 sncRNAs which were not reported before. This was the first study of genome-scale identification of sncRNAs for a basidiomycete. The sequence length of sncRNAs accounted for 0.054% of CCEF00389 genome, and the identified sncRNAs included most classes of known sncRNAs. However, the snRNA U1 was not identified not only in CCEF00389, but also in other basidiomycetous genomes. This implies that if snRNA U1 of basidiomycetes exists, it has a sequence that varies significantly from other organisms.

For some sncRNAs, the position of loci may be associated with some potential functions. The UTR regions of the CCEF00389 genome assembly were not precisely determined, so we calculated the distances of sncRNAs to the gene boundary (start/stop codon) for possible location in pseudo-UTR regions. The snRNAs and tRNAs had a higher possibility to be located in pseudo-UTR regions, while the miRNAs were more common in introns.

There were 197 sncRNAs in CCEF00389 genome, which had detectable homologues in another strain of* P. ostreatus*, and 74 sncRNAs in CCEF00389 genome which were also found in some other Agaricomycotina fungi. However, only 15 sncRNAs in CCEF00389 genome had homologues in* Ustilago maydis*, which does not belong to Agaricomycotina. It suggests that most sncRNAs of* P. ostreatus *were not conserved across Agaricomycotina fungi.

Long ncRNA (lncRNA) is also a kind of impressive ncRNA which plays critical roles in multiple biological processes based on diverse underlying mechanisms [17, 22]. And prediction of the interaction between ncRNAs and proteins has attracted much attention because the ncRNAs function mediated with proteins. In the future work, we will focus on identification and analysis of lncRNAs [12] and prediction of the interactions between ncRNAs and proteins [72–74].

## Supplementary Material

Supplemental Table 1: Comparison of genomic features for edible fungi. Supplemental Table 2: General information of tRNAs in the genome assembly of CCEF00389. Supplemental Table 3: Location of tRNAs in the genome assembly of CCEF00389. Supplemental Table 4: Identities of matches of tRNA between *P. ostreatus* CCEF00389 and some other Agaricomycotina fungi. There are 57 of them highly conserved among Agaricomycotina fungi.

## Figures and Tables

**Table 1:** General features of the *P. ostreatus* CCEF00389 genome assembly.

Number of scaffolds	2,529
Length of all scaffolds combined (Mb)	35.8
GC content (%)	49.54
Scaffold N50 value (bp)	394,787
Number of large scaffolds (>1000)	794
Length of large scaffolds (Mb)	34.9
Number of protein-coding genes	13,438

**Table 2:** Genomic loci, distance to gene boundary, and neighboring gene of sncRNAs identified using Rfam.

ID	Location	Strand	Distance to gene boundary	Neighbor
U2.1	scaffold37_107912–108102	−	470	g12507
U2.2	scaffold43_54422–54612	−	487	g12899
U2.3	scaffold43_63239–63429	+	536	g12905
U2.4	scaffold37_118109–118299	+	329	g12510
RNase_MRP.1	scaffold1_325685–326108	+	172	g8330
U6.1	scaffold_59_650694–650810	+	315	g7427
U6.2	scaffold_80_192511–192627	+	150	g8186
U6.3	scaffold63_39552–39668	+	399	g4097
U5.1	scaffold16_469793–469909	−	959	g10739
snoZ13_snr52.1	scaffold46_213325–213430	−	394	g13123
U4.1	scaffold113_43830–43975	+	459	g5145
snoZ13_snr52.2	scaffold1_729232–729339	−	Intron	g8490
snoZ13_snr52.3	scaffold46_213071–213178	−	646	g13123
snosnR60_Z15.1	scaffold_75_65986–66075	−	Intron	g7941
Hammerhead_3.1	scaffold59_142237–142291	+	277	g3917
Hammerhead_3.2	scaffold59_152597–152651	−	257	g3922
Hammerhead_3.3	scaffold_12_734778–734832	+	262	g1296
Hammerhead_3.4	scaffold59_146486–146540	+	685	g3919
SNORD24.1	scaffold60_67979–68061	−	Intron	g3964
Afu_455.1	scaffold_11_316838–316924	−	Intron	g963
Hammerhead_3.5	scaffold25_333837–333891	+	Intron	g11689
SNORD46.1	scaffold37_225673–225759	+	1031	g12558

**Table 3:** Genomic loci, distance to gene boundary, and neighboring gene of miRNAs.

ID	Location	Strand	Distance to gene boundary	Neighbor
miR-124-5p	scaffold_29_13029–13047	+	Intron	g3043
miR-190a-3p	scaffold48_55792–55810	+	53	g13155
miR-788-5p	scaffold8_754188–754207	−	Intron	g9721
miR-383-3p	scaffold_80_111549–111567	−	Intron	g8156
miR-466g	scaffold8_19655–19673	−	94	g9475
miR1171	scaffold_70_119604–119626	+	2094	g7800
miR-467g	scaffold_26_54067–54085	−	147	g2750
miR-190a-3p	scaffold48_55792–55810	+	53	g13155
miR1427	scaffold85_42007–42025	−	110	g4652
miR2095-5p	scaffold_29_43092–43110	+	1121	g3054
miR-788	scaffold8_754188–754207	−	Intron	g9721
miR2673a	scaffold32_244540–244559	+	71	g12059
miR2673a	scaffold25_190886–190904	+	Intron	g11628
miR2673a	scaffold_15_223801–223819	−	Intron	g1786
miR2673b	scaffold32_244540–244559	+	71	g12059
miR2673b	scaffold25_190886–190904	+	Intron	g11628
miR2673b	scaffold_15_223801–223819	−	Intron	g1786
miR-2709	scaffold64_62205–62223	+	Intron	g4162
miR-2783	scaffold392_645–663	+	Intron	g5776
miR-190a-3p	scaffold48_55792–55810	+	53	g13155
miR156h-3p	scaffold151_19717–19735	−	Intron	g5461
miR4243	scaffold_15_464570–464589	−	Intron	g1895
miR-3677-5p	scaffold61_171654–171672	−	363	g4080
miR-3775	scaffold1170_174–192	−	Intron	g6228
miR3948	scaffold155_16079–16098	+	3715	g5479
miR3948	scaffold8_7775–7794	−	Intron	g9470
miR-4459	scaffold7_14624–14642	+	Intron	g9172
miR-4968-3p	scaffold_14_64946–64966	−	Intron	g1539
miR-4968-3p	scaffold34_263134–263153	−	Intron	g12318
miR-4968-3p	scaffold_24_6521–6540	−	Intron	g2572
miR-4968-3p	scaffold3_287008–287026	+	Intron	g8829
miR-5352-5p	scaffold53_49526–49544	−	Intron	g13380
miR-5455-3p	scaffold54_13491–13509	−	Intron	g3643
miR-6012-5p	scaffold14_172648–172666	−	Intron	g10243
miR6214	scaffold58_68922–68940	−	Intron	g3823
miR-6606-5p	scaffold_19_158806–158825	+	Intron	g2084
miR-7426-5p	scaffold46_187269–187287	+	Intron	g13115
miR7734-3p	scaffold21_198083–198101	+	Intron	g11335
miR-190a-3p	scaffold48_55792–55810	+	53	g13155
miR-8481-5p	scaffold1214_35–53	+	Intron	g6248
miR-8922	scaffold21_9675–9693	+	Intron	g11273
miR-8986a	scaffold_4_231055–231073	−	Intron	g184
miR-9189b	scaffold_24_289739–289757	−	Intron	g2662
miR-9400-5p	scaffold3_354824–354842	+	Intron	g8861
miR-190a-3p	scaffold48_55792–55810	+	53	g13155
miR9773	scaffold390_1391–1409	−	292	g5774
